# Testicular seminoma: clinical, imaging, and histologic features in nine horses

**DOI:** 10.1093/jvimsj/aalag129

**Published:** 2026-07-06

**Authors:** Bridget Ratliff, Isabelle Kilcoyne, Pouya Dini, Scott A Katzman, Mitja Miklavcic, Melissa Chromik, Eunju Choi, Betsy Vaughan

**Affiliations:** Comstock Equine Hospital, Reno, NV, United States; Department of Surgical and Radiological Sciences, School of Veterinary Medicine, University of California-Davis, Davis, CA, United States; Department of Population Health and Reproduction, School of Veterinary Medicine, University of California-Davis, Davis, CA, United States; Department of Surgical and Radiological Sciences, School of Veterinary Medicine, University of California-Davis, Davis, CA, United States; Department of Surgical and Radiological Sciences, School of Veterinary Medicine, University of California-Davis, Davis, CA, United States; Department of Pathology, Microbiology, and Immunology, School of Veterinary Medicine, University of California-Davis, Davis, CA, United States; Department of Pathology, Microbiology, and Immunology, School of Veterinary Medicine, University of California-Davis, Davis, CA, United States; Department of Surgical and Radiological Sciences, School of Veterinary Medicine, University of California-Davis, Davis, CA, United States

**Keywords:** cryptorchid, horse, seminoma, testicle, weight loss

## Abstract

**Background:**

Testicular seminomas in horses are infrequently reported in the veterinary literature and long-term survival and metastatic potential are not well documented.

**Hypothesis/Objectives:**

Describe the clinical, imaging, and histologic features in 9 horses with testicular seminoma.

**Animals:**

Medical records of 9 horses diagnosed with testicular seminoma at a referral hospital between 2000 and 2024 were reviewed.

**Methods:**

Horses were included if seminoma was confirmed on histopathology from biopsy or necropsy. Information retrieved included signalment, history, clinical pathology, ultrasonographic findings, treatment, and outcome.

**Results:**

Median age was 18 years (range, 8-26). Three of 9 horses presented for systemic disease (colic, 2; ventral edema, 1). All 3 had large abdominal masses palpable on rectal examination and were euthanized after diagnostic evaluation. Six horses presented for unilateral testicular enlargement (5) or cryptorchid castration (1). These horses underwent castration and the removed testes had features of malignancy with lymphatic neoplastic emboli. Five of 6 horses were euthanized between 4 months and 3 years after castration because of acute weight loss and abdominal masses identified on rectal examination or ultrasonographic examination, attributable to metastatic spread. One additional horse was euthanized for unrelated reasons.

**Conclusions and clinical importance:**

Horses presented with testicular enlargement and diagnosis of testicular seminoma should undergo diagnostic screening, including transabdominal or transrectal ultrasonographic examination to determine the presence of possible metastasis. Negative screening results at the time of diagnosis do not rule out the possibility of metastasis, and frequent screening at regular intervals is prudent in such cases. Horses with testicular seminoma of retained or descended testicles have a poor long term prognosis because of high potential for metastatic spread.

## Introduction

Testicular tumors are rarely reported in horses, representing approximately 0.04%-0.9% of all tumors in horses.^1^ The true incidence is difficult to determine because most male horses are castrated at a young age, and excised testicular tissue is rarely examined histologically.^2^ Primary testicular tumors are divided into germinal and nongerminal types, with germinal neoplasms arising from germ cells of the seminiferous epithelium and nongerminal tumors arising from the testicular stromal cells.^3^ Seminomas arise from the germ cells of the seminiferous tubules and are the most common testicular neoplasm in mature horses.^1^^,^^3^^,^^4^ In horses, multiple reports suggest that seminomas occur more frequently in cryptorchid testes, but a definitive association is difficult to establish because of limited case numbers and incomplete population-level data.^2^^,^^4–7^

The most commonly reported clinical finding associated with testicular neoplasia in horses is testicular enlargement.^5^^,^^6^^,^^8^^,^^9^ Testicular tumors must be differentiated from other causes of testicular or scrotal enlargement such as orchitis, epididymitis, hydrocele, varicocele, hematoma, scrotal hernia, and spermatic cord torsion.^3^ Ultrasonography is a useful diagnostic tool in these cases, but the ultrasonographic features reported for seminomas, including testicular enlargement, loss of normal testicular architecture, and heterogeneous echotexture are nonspecific and overlap with other testicular neoplasms and inflammatory conditions.^5^^,^^9^ Castration is recommended when testicular neoplasia is suspected, and histopathologic examination is necessary for a definitive diagnosis.^6^^,^^10^ Histologically, seminomas frequently exhibit features consistent with malignancy, including cellular atypia and increased mitotic activity.^4^^,^^9^ However, many case studies do not provide long-term follow-up after castration, and therefore, metastatic potential has not been correlated to histologic changes.^6^^,^^9^ Seminomas have been shown to have metastatic potential in horses,^11–13^ with reported metastases to the lumbar lymph nodes,^14^ kidney,^11^ heart, and lungs.^8^ Consequently, metastatic testicular seminoma may be associated with a variety of clinical presentations including colic,^7^ acute weight loss,^15^ or respiratory signs.^8^

Our objective was to describe the presenting complaints, clinicopathologic and imaging findings, and outcome in horses with a definitive diagnosis of testicular seminoma on histopathologic examination. We hypothesized that testicular seminomas in horses have metastatic potential and that histopathologic features of malignancy are associated with worse long-term outcomes.

## Materials and methods

The primary inclusion criterion for our study was a histopathologic diagnosis of testicular seminoma established by biopsy or necropsy. Computerized medical records from the William R. Pritchard Veterinary Medical Teaching Hospital (VMTH) at the University of California-Davis School of Veterinary Medicine were reviewed for the period between January 1st, 2004 and December 31st, 2024, and cases meeting this criterion were included.

Data retrieved from the medical record included signalment, history, presenting complaint, presenting clinicopathologic data, ultrasonographic findings, treatment, histopathologic findings, and outcome. Short-term survival was defined as survival to hospital discharge. For horses that were euthanized, results of the necropsy examination were recorded. Long-term survival was defined as survival for 12 months or longer after hospital discharge. Long-term follow-up information was obtained either through review of medical records or through a standardized telephone questionnaire. Owners were asked whether the horse was alive at the time of follow-up, and if not, whether death was a result of euthanasia or natural causes, the timing of death, the reason for euthanasia, and whether necropsy findings were available.

### Ultrasonographic examination

Horses underwent all or a combination of testicular, transabdominal, and transrectal ultrasonographic examination. Testicular ultrasonography involved evaluation of the scrotum, testes, and epididymides. Abdominal ultrasonography included evaluation of the kidneys, liver, spleen, stomach, duodenum, small intestine, cecum, cecal mesentery, large colon, and peritoneal cavity. Transrectal ultrasonography included the terminal aorta, internal and external iliac arteries, and local lymph nodes. Ultrasonography was performed using one of several ultrasound machines available during the long study period: Biosound Technos (Biosound Technos, Universal Ultrasound, 299 Adams Street, Bedford Hills, NY 10507), Esaote Biosound Twice (Biosound Twice, Vetel Diagnostics, 4850 Davenport Creek Rd, San Luis Obispo, CA 93401), Esaote MyLab Seven (Esaote MyLab Seven, Esaote North America, Inc., 11907 Exit 5 Pkwy, Fishers, IN 46037), or Esaote MyLab ×8 (Esaote MyLab ×8, Esaote North America, Inc., 11907 Exit 5 Pkwy, Fishers, IN 46037) ultrasound machines. Horses were lightly sedated with detomidine HCL (0.004-0.015 mg/kg IV) with or without butorphanol tartrate (0.01-0.02 mg/kg IV) when needed.

### Histopathologic analysis

Identified biopsy and necropsy cases were obtained from the pathology archives and reviewed by 2 authors. Presence of vascular or lymphatic neoplastic emboli were considered features of malignancy. In cases where necropsy was performed, effacement of normal tissue by the neoplasm also was considered a feature of malignancy. Mitotic count was performed in 2.37 mm^2^ for each biopsy and necropsy case.

### Statistical analysis

Data were summarized and descriptive statistics were performed. Results are reported as medians and ranges (Excel version 2409; Microsoft Corp).

## Results

Nine horses with testicular seminoma confirmed on histopathologic examination met the inclusion criteria. The study population included 5 stallions with bilaterally descended testes and 4 cryptorchid horses. The median age was 18 years (range, 8-26 years). Represented breeds included Quarter Horses (3; 33%), Arabians (2; 22%), and 1 horse each (11%) of Thoroughbred, Lusitano, Friesian, and Azteca pony.

Five horses (56%) were presented for unilateral testicular enlargement, with onset of enlargement ranging from 5 days to 3 years. Of these 5 horses, 3 were presented with enlargement of the left testis and 2 with enlargement of the right testis. Of the 4 cryptorchid horses, only 1 was presented for cryptorchid evaluation and subsequently was diagnosed with a right abdominally retained testis (case 4).

The 3 remaining cryptorchid horses were presented for signs of systemic disease, including 2 horses with acute colic (<12-h duration) and 1 horse that presented for evaluation of ventral edema of 3-day duration. All 3 horses were euthanized after initial diagnostic evaluation and subsequently were found to have abdominally retained testes (1 right testis [case 1], 1 left testis [case 3], and 1 with bilaterally retained testes [case 5]). Before necropsy, cases 1 and 5 were believed to be geldings. Case 3 was a known cryptorchid and was being actively used as a breeding stallion.

### Presenting clinicopathological data

All horses presented for evaluation of systemic disease had increased heart rates (range, 48-60 beats per minute [bpm]) and 1 horse (case 1) had a left-sided systolic heart murmur. All other vital signs were within normal limits except for 1 horse (case 7) presented for evaluation of testicular enlargement with a rectal temperature of 101.7°F.

Rectal palpation was performed in both horses presented for colic (cases 1 and 5) and a large caudal abdominal mass was palpated in both cases.

### Hematologic variables

Hematologic data are presented in Table 1. Results were available for 4 of the 9 horses on initial presentation. One horse that was presented for evaluation of colic (case 5) had an increased white cell count (13 000 cells/μL; reference range, 5000-11 600 cells/μL). Two horses underwent abdominocentesis. One horse that was presented for evaluation of colic (case 5) had normal peritoneal fluid cytology. Peritoneal fluid cytologic analysis from another horse that presented with ventral edema (case 3) showed pleomorphic nucleated cells with a high nuclear:cytoplasmic (N:C) ratio and rare mitotic figures, consistent with the presence of neoplastic cells.

**Table 1:** Presenting hematology and serum biochemistry data in horses presented for testicular seminoma between 2004 and 2024.

**Case**	**WBC (per uL)**	**Fib (mg/dL)**	**TP (g/dL)**	**Calcium (mg/dL)**	**Creatinine (mg/dL)**	**BUN (mg/dL)**
**2**	9 590	600	7.5	20.4	3.5	79
**5**	13 000	200	7.7	12.3	0.8	22
**6**	6 360	400	6.8	11.8	1.5	21
**7**	8 740	200	6.2	12.6	1.6	20
**9**	9 500	200	7.1	12.8	1.5	16

Abbreviations: BUN = blood urea nitrogen; Fib = fibrinogen; TP = total protein; WBC = white blood cell.

One horse (case 2) had blood testing performed when it was re-presented for acute weight loss 4 months after initial presentation that included markedly increased serum total calcium (20.4 mg/dL; reference range, 11.4-14.1 mg/dL) and creatinine (3.5 mg/dL; reference range, 0.9-2.0 mg/dL) concentrations.

### Ultrasonography

All horses presented for testicular enlargement with subsequent castration (*n* = 5) had bilateral testicular ultrasonography and a heterogenous appearance of the abnormal testicle was noted in all (cases 2 and 6-9). Three cases had additional transabdominal and transrectal ultrasonography or both at the time of initial presentation. One horse (case 8) had a chain of enlarged heterogenous lymph nodes adjacent to the terminal aorta on the affected side identified on transrectal ultrasonography. The other 2 horses (cases 6 and 9) had no abnormal findings.

**Figure 1:**
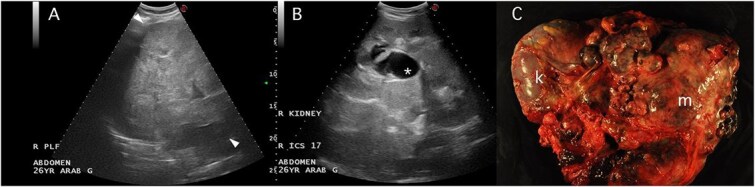
(A) Large abdominal seminoma in a 26-year-old Arabian hemi-castrated stallion (arrowheads). This mass extended from the right kidney to the bladder. Image obtained with a 5 MHz curvilinear transducer at a depth of 27.9 cm. Dorsal is to the right. (B) Associated hydronephrosis of the right kidney (asterisk) due to ureteral compression by the mass. Image obtained with 5.0 MHz curvilinear transducer at a depth of 21.1 cm. Dorsal is to the right. (C) Gross image of the retroperitoneal mass (m) that surrounds the caudal vena cava, descending aorta, ureters, adrenal glands, and kidneys (k). The mass is tan to black, multinodular, and firm.

**Figure 2:**
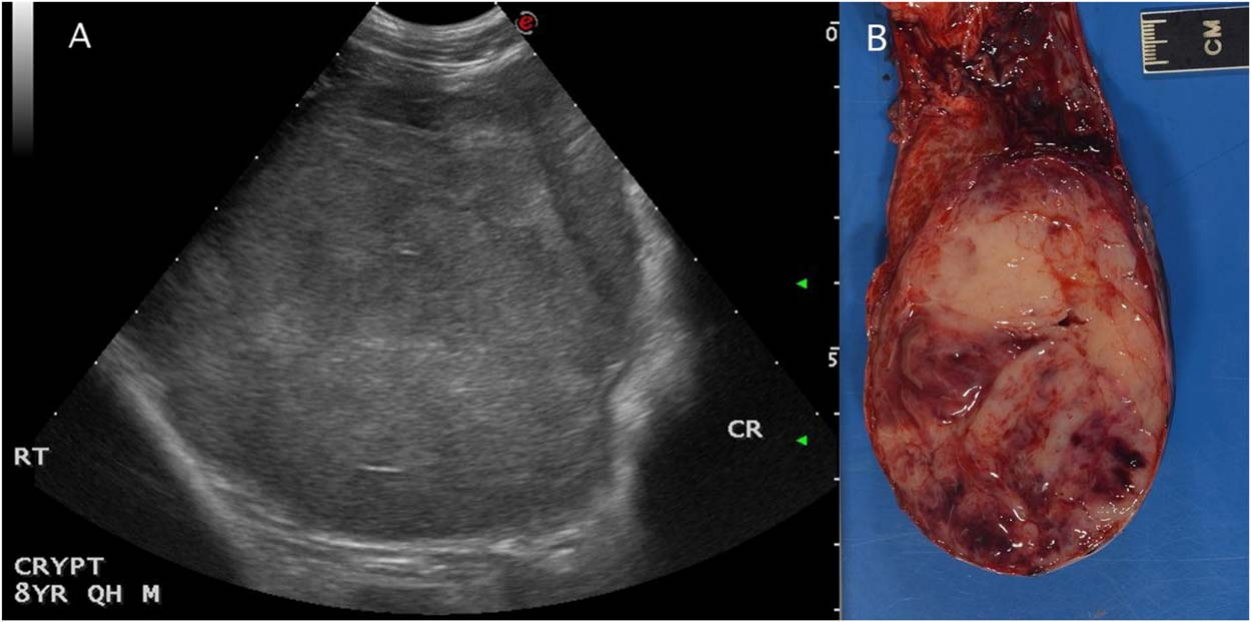
(A) Abdominal right testis in an 8-year-old QH stallion presenting for cryptorchid castration. Image obtained with a 7.5 MHz curvilinear microconvex transducer at a depth of 9.1 cm. Cranial is to the right. (B) Gross image of a bisected right testicle submitted for biopsy and diagnosed with a seminoma. Within the testicle, there are multiple, coalescing, soft, pale tan masses with multifocal areas of hemorrhage.

Of the horses that were castrated, 2 underwent abdominal ultrasonography 4 (case 2) and 9 (case 8) months later because of acute weight loss. Both had a large caudal abdominal mass adjacent to the kidney on the same side as the castrated affected testicle. Additional abnormalities included hydronephrosis and hydroureter with dilated mesenteric vasculature secondary to extraluminal compression by the large intra-abdominal mass (case 2, Figure 1) and mild small intestinal wall thickening considered to be an incidental finding (case 8). One horse was presented as a known cryptorchid (case 4) and had transabdominal ultrasonography that only identified a heterogenous abdominally retained testicle (Figure 2).

**Figure 3:**
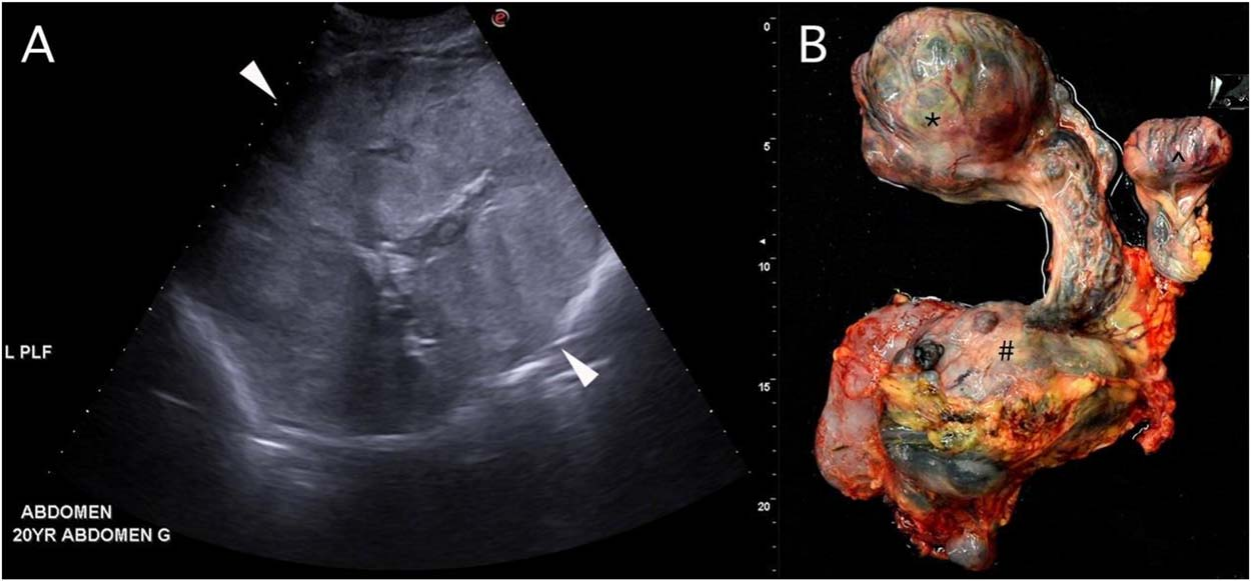
(A) Abdominal left testis in a 20-year-old Azteca horse presumed to be a gelding presenting for acute colic. Image obtained with a 5 MHz curvilinear transducer at a depth of 23.1 cm. Cranial is to the right. (B) Gross image of the left testicle (*), right testicle (^), and retroperitoneal mass (#) from necropsy. Seminomas are present in both testicles with metastasis to the retroperitoneal space.

**Figure 4:**
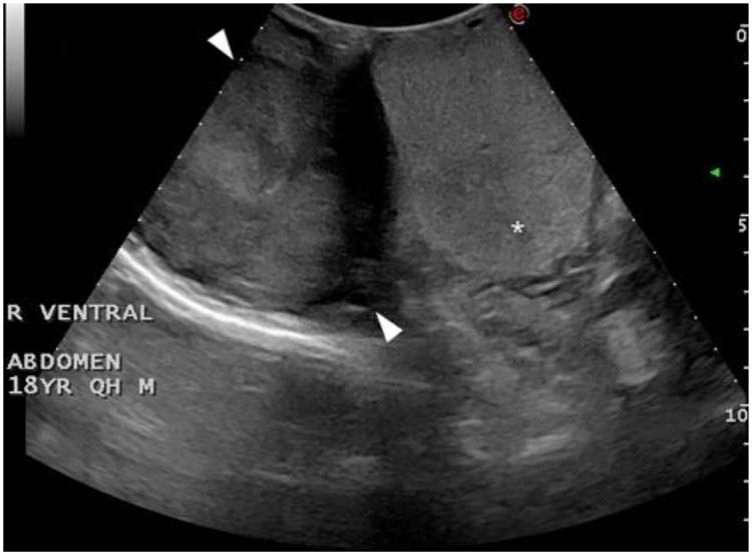
Seminoma in the left testis of a 18-year-old quarter horse breeding stallion adjacent to the normal right testis (asterisk). Image obtained with a 5 MHz curvilinear transducer at a depth of 13.8 cm. Lateral is to the right.

Two of the 3 horses that initially were presented for systemic disease had transabdominal ultrasonography (cases 3 and 5) that identified a large paralumbar mass in both (Figure 3). Case 3 had additional space-occupying lesions of the liver, spleen, and left kidney with thickened peritoneum and severe peritoneal effusion. All masses were similar in appearance, with a round to oval shape and heterogeneous echogenicity. Intra-abdominal masses ranged in width from 8.8 to 30 cm (median, 15.5 cm), length from 8.2 to 18 cm (median, 13.1 cm), and depth from 7.0 to 21.3 cm (median, 17.6 cm) (Figure 1). Abnormal testicles ranged in width from 8.8 to 16.5 cm (mean, 12.7 cm; median, 12.7 cm), length from 5.8 to 9.6 cm (median, 6.5 cm), and depth from 4.7 to 12.3 cm (median, 8.3 cm; Figure 4).

### Surgery

Six horses underwent castration under general anesthesia. Two horses (cases 2 and 9) underwent bilateral castration. Three horses (cases 6-8) underwent unilateral castration of the affected testis. The 5 horses with bilaterally descended testicles underwent castration with primary closure of the surgical incisions. One cryptorchid (case 4) underwent laparoscopic removal of the abdominally retained testis. The castration history of this horse was unknown, but a surgical scar was identified during preparation for surgery, indicating previous removal of the descended testis. All abnormal testicles were submitted for histopathology. In 1 bilateral castration (case 2), the grossly normal testicle also was submitted.

All horses that underwent castration initially were treated perioperatively with injectable systemic antibiotics and nonsteroidal anti-inflammatory drugs (NSAIDs) for 24 h (procaine penicillin [22,000 IU/kg IM q12h], gentamicin [6.6 mg/kg IV q24h], and flunixin meglumine [1.1 mg/kg IV q12h]). Five of 6 horses were transitioned to PO antibiotics (trimethoprim sulfadiazine; 30 mg/kg PO q12h) and a tapering PO dose of flunixin meglumine. The duration of PO antibiotic administration was 7 days in horses that did not develop complications (cases 4 and 8) and ranged from 10 to 24 days in the other cases (cases 2, 6, and 9). One horse (case 7) continued IV antibiotics and NSAIDs for 4 days and did not receive PO medications.

Three of the 6 horses that underwent castration developed complications. Two horses (cases 2 and 6) developed moderate scrotal swelling the day after surgery. A hematoma was diagnosed on ultrasonographic examination in 1 horse (case 6). One horse (case 9) sustained a fracture of the tuber coxae during recovery from anesthesia and subsequently developed contralateral limb laminitis.

### Histopathologic findings of biopsied testes

Six horses (cases 2, 4, 6-9) had an abnormal testis removed and submitted for histopathology.

Grossly, all affected horses had similar features on cut sections, described as single to multiple, pale tan to pink, soft coalescing nodules that variably bulged (Figure 2). Case 8 had 2 additional, grossly similar discrete masses within the tunica vaginalis that were 5 and 6 cm in diameter. For case 9, 95% of the testicle was estimated to be effaced by a mass that was 12 cm × 11 cm × 9 cm.

Histologic features included sheets and lobules of round neoplastic cells with distinct cell borders, a moderate amount of eosinophilic cytoplasm, and a round, central nucleus with vesiculated to coarsely granular chromatin with a large central magenta nucleolus separated by fine fibrovascular stroma, occasionally surrounding remnant degenerate seminiferous tubules. Anisocytosis and anisokaryosis ranged from moderate to marked and multinucleated cells were frequent throughout the neoplastic cell population. The mitotic count ranged from 23 to 64 mitotic figures in 2.37 mm^2^ (average approximately 37 mitoses). Individual cell necrosis or apoptosis was identified in 4 of the cases (cases 2, 7-9), whereas larger areas of necrosis were seen in 2 of the cases (cases 4 and 6). Lymphatic neoplastic emboli were a common feature in all cases. The contralateral testicle of case 2 had hemorrhage with hemosiderophages, moderate fibrosis, interstitial cell hyperplasia, mild, multifocal lymphocytic interstitial orchitis, and mild hypospermia. The contralateral testicle of case 9 was not submitted for histopathologic examination.

### Outcome

The median number of days hospitalized postsurgery was 4 days (range, 1-14 days).

The outcome is summarized in Table S1. All 6 horses were discharged after castration. Both horses that presented for colic were euthanized at the time of initial presentation because of persistent pain and poor prognosis associated with suspected neoplasia, based on identification of caudal abdominal masses palpated per rectum (cases 1 and 5) and visualized on ultrasonography (case 5). The horse that was presented for ventral edema (case 3) had evidence of multiple masses throughout the abdomen on ultrasonographic examination (inguinal, liver, and spleen) in addition to neoplastic cells identified on peritoneal fluid cytology, and was also euthanized.

Of the 6 horses discharged from the hospital, 4 were alive at 12 months postdischarge, but none of these horses was alive 3 years after discharge. One horse (case 6) was euthanized for an unrelated issue (lameness) 3 years after discharge. The horse was reported to be of normal body condition at that time, with no other clinical signs associated with the previous neoplasm removal. No necropsy findings were available. The remaining 5 horses developed substantial weight loss over the course of approximately 1 month, between 4 months and 3 years (median, 13.5 months) after discharge. Four of these horses (cases 2, 4, 7, and 8) underwent further diagnostic evaluation and the presence of abdominal masses was identified in all cases on rectal palpation, transabdominal ultrasonographic examination, or both. They were subsequently euthanized because of suspicion of metastatic seminoma. Necropsy results were available for 2 horses (cases 2 and 7). The remaining horse (case 9) was euthanized with no further evaluation.

### Gross necropsy findings

All horses euthanized in the hospital (*n* = 5; cases 1-3, 5, and 7) were necropsied. Two cases (cases 2 and 7) previously had been diagnosed with unilateral testicular seminoma and subsequently were re-presented for signs attributable to metastases and were euthanized.

Necropsy was performed on 5 horses (cases 1-3, 5, and 7). Of these, 2 horses were unilateral cryptorchids (cases 1 and 3) and 1 horse (case 5) was bilaterally cryptorchid. Two horses (cases 1 and 3) had widespread involvement of the visceral serosa of multiple organs. Grossly, the masses were characterized as pale tan to dark purple, soft, multinodular, and bulged on section. In all cases, a mass was present in the retroperitoneal or sublumbar region and entrapped organs locally. In 3 horses (cases 1, 2, and 5), the neoplasm incarcerated major vessels, including the abdominal aorta and caudal vena cava, along the dorsal body wall. Similarly, in most horses (cases 2, 3, 5, and 7), the neoplasm entrapped the kidney and ureter, resulting in variable degrees of hydronephrosis (Figure 1). Lymph node metastasis was suspected in all cases but could not be confirmed histologically because of the lack of remnant lymphoid tissue. This finding may imply complete effacement of lymph node by metastasis or represent non-lymph node-related serosal metastasis. Thoracic metastasis was confirmed in case 3 where the perithymic fat was affected. Thoracic metastasis involving lymph nodes was suspected in cases 1 and 7 but was not confirmed histologically. Metastasis within visceral organs was not identified.

### Histopathological necropsy findings

In all 5 cases, the neoplasm was histologically similar to the cases that were biopsied and diagnosed as malignant seminoma. In the 3 cases with retained testicles (cases 1, 3, and 5), the intra-abdominal masses were presumed to be the retained testicle, although no normal tissue architecture was present to confirm this suspicion.

When present and recognizable, both testicles were examined histologically. The contralateral testis of case 1 had a seminoma. Bilateral seminomas were present in 1 case with bilateral cryptorchidism (case 5). In cases 3 and 7, the right testicle was descended but atrophied and degenerative with no histologic evidence of neoplasia, but in case 7, the descended right testicle had chronic bacterial orchitis and epididymitis.

Four of the cases (1-3 and 7), had substantial myocardial fibrosis with occasional degeneration and myocyte loss. Cases 1-3 had entrapped major abdominal vessels associated with abdominal masses, which may have caused cardiovascular compromise. Case 7 had nodules identified in the pericardium grossly that may have contributed to cardiac changes.

The mitotic count for necropsy cases ranged from 21 to 61 in 2.37 mm^2^ (average, 36.8).

## Discussion

Testicular seminoma demonstrated metastatic potential in the horses included in our study, supporting our first hypothesis. This finding is consistent with published case series describing metastasis of testicular seminoma to locations such as abdominal organs and the thoracic cavity.^8^^,^^11^^,^^13^^,^^16–18^

All abnormal testes (*n* = 6) submitted for histopathology had features of malignancy including vascular or lymphatic neoplastic emboli or both. Mitotic count was variable between 21 and 64 mitoses in 2.37 mm^2^. At long-term follow-up, 5 of 6 horses had been euthanized because of signs attributable to metastatic spread with confirmed evidence on necropsy in 2 cases (cases 2 and 7) and clinical evidence of metastasis in 2 others based on palpation of a caudal abdominal mass on rectal examination (cases 4 and 8). This finding supports our second hypothesis that histopathologic evidence of malignancy is associated with poor long-term outcome. Evidence of metastasis warrants a guarded prognosis for long-term survival in horses diagnosed with primary seminoma.

Previous reports identified systemic metastasis of testicular seminoma within 2 years of initial diagnosis.^7^^,^^8^^,^^13^ We found a similar time frame with a median of 13.5 months until repeat presentation for systemic compromise (range, 4 months to 3 years) and subsequent identification of metastases.

Acute weight loss was the most common presenting complaint in horses presented with metastasis (*n* = 5) after previous castration and diagnosis of seminoma. Colic (*n* = 2) and ventral edema (*n* = 1) also were noted in horses presented with systemic signs before diagnosis of metastatic testicular seminoma. Although metastatic seminoma is uncommon, the combination of recent weight loss or colic, and identification of a caudal abdominal mass during diagnostic evaluation in a male horse should alert the clinician to metastatic seminoma as a differential diagnosis.

Transabdominal and testicular ultrasonographic examination provided the most valuable diagnostic information in our cases. Once systemic signs of disease were present, caudal abdominal masses were identified in all cases where ultrasonography was performed. At the time of presentation for testicular enlargement, ultrasonographic evaluation identified testicular masses in all affected horses. Transrectal ultrasonography was performed in 3 horses at initial presentation, but only 1 horse had abnormalities identified associated with the sublumbar lymph nodes.

Given the observed frequency of metastatic disease in this cohort, it would seem prudent to perform transrectal and transabdominal ultrasonography to evaluate for the presence of additional masses in a horse with suspected testicular neoplasia. In a study of 143 human patients with testicular germ cell tumors, 10%-30% of seminoma metastases were occult and not observed on computed tomography (CT) imaging.^19^ Because of the lower sensitivity of ultrasonography and radiology compared with CT imaging as a screening tool, it is likely that a higher incidence of metastases could be missed in affected horses. Transabdominal and transrectal ultrasonography, however, still is recommended in suspected cases of testicular neoplasia, particularly seminoma, to rule in the presence of metastasis. The clinician should be aware that normal systemic screening at the time of presentation for testicular seminoma in horses does not exclude metastatic disease, and periodic screening in the subsequent months is prudent in such cases.

In canine and human patients, an association between cryptorchidism and development of testicular cancer has been reported,^20^^,^^21^ with up to a 5% risk of neoplasia in patients with intra-abdominal testes.^22^ Orchidopexy has been shown to lower the risk of testicular cancer when performed before puberty, suggesting that moving the testis into the scrotum decreases the risk of testicular cancer.^23–26^ However, this procedure has not been routinely performed in horses.

Routine early castration in horses has been hypothesized as an explanation for the low incidence of testicular neoplasia in the equine population.^2^ In our study, the youngest horse diagnosed with seminoma was an 8-year-old cryptorchid. Early castration of known cryptorchids should be performed to avoid the risk of potential malignant transformation. In cases with suspected testicular neoplasia, we recommend closed castration with removal of as much of the spermatic cord and tunica vaginalis as possible to increase the chance of obtaining clean margins. It should be noted, however, that in our case study all horses with testicular seminomas that were castrated had lymphatic neoplastic emboli.

Limitations of our study are inherent to retrospective analysis. Retrieval of data was limited by the completeness of the medical record. Bias associated with diagnostic tests performed, case management, and clinical judgment of the attending clinician could not be controlled. The small number of cases presented over a long period and the 20-year duration of the study may have contributed to substantial variability associated with changes in personnel and advancements in diagnostic testing.

In conclusion, testicular seminoma demonstrated metastatic potential in our case series and was associated with poor long-term prognosis. Histologic vascular or lymphatic neoplastic emboli should raise concern for potential metastatic disease. Diagnostic evaluation to determine the possible presence of metastasis at the time of presentation should include rectal examination and a combination of transabdominal and transrectal ultrasonography. Absence of detectable abnormalities at initial evaluation does not preclude metastatic disease, and continued surveillance at regular intervals after diagnosis is prudent.

## Supplementary Material

Supplementary_table_1_aalag129

## Off-label antimicrobial declaration

The authors declare no off-label use of antimicrobials.

## Institutional animal care and use committee or other approval declaration

The authors declare no institutional animal care and use committee or other approval was needed.

## Abbreviations

CT: computed tomography
VMTH: Veterinary Medical Teaching Hospital
