# Supplementary material for: Testicular seminoma: clinical, imaging, and histologic features in nine horses
Source: J Vet Intern Med. 2026 Jul 6;40(4):aalag129. doi: 10.1093/jvimsj/aalag129 (PMC13336631; doi:10.1093/jvimsj/aalag129)
Supplement: Supplementary_table_1_aalag129 [file supplementary_table_1_aalag129.docx]

Supplementary Table 1: Presenting clinicopathological data, diagnostic findings, treatment and outcome of horses diagnosed with testicular seminoma.

|  | **Signalment** | **Presenting Complaint** | **Ultrasonographic Impressions** | **Treatment** | **Outcome (Complications) Necropsy Findings** | **Histopathology** |
| --- | --- | --- | --- | --- | --- | --- |
|  |  |  |  |  |  |  |
| **Case 1** | 25yo Arab G* | Colic |  | None | **Euthanized**  Necropsy: large abdominal mass enveloping great vessels and intestines. Metastasis to sublumbar LN, spleen, liver, body wall and diaphragm. |  |
| **Case 2** | 26yo Arab  S | R testicle enlargement | Heterogenous and enlarged right testicle. | Bilateral castration | **Discharged** 4 days after surgery (moderate scrotal swelling).  **Euthanized** 4 months later due to acute weight loss and abdominal mass on ultrasound  Necropsy: large abdominal mass incarcerating great vessels and kidneys/ureters. Metastasis to sublumbar lymph nodes. | 11 mitoses observed in ten high powered 400x fields. Anisocytosis and anisokaryosis was moderate. Lymphatic invasion. No neoplastic cells within the epididymis or surgical margin. |
| **Case 3** | 18yo QH  Unilateral Crypt | Ventral edema | Large left inguinal mass. Heterogenous liver and spleen. Disrupted left kidney with dilated renal pelvis. Marked free peritoneal fluid. | None | **Euthanized**  Necropsy: large abdominal mass entrapping left ureter. Metastasis to kidneys, spleen, liver, sublumbar lymph nodes and caudal mediastinum. Tumor emboli in numerous vessels and renal tubules. |  |
| **Case 4** | 8yo QH  Unilateral Crypt | Crypt castration | Presumed right abdominally retained testicle with heterogenous appearance. | Laparoscopic R crypt orchidectomy | **Discharged** 1 day after surgery  **Euthanized** 3 months later due to acute weight loss and abdominal mass on rectal ultrasound | 27 mitoses observed in ten high powered 400x fields. Anisocytosis and anisokaryosis was moderate to marked. Venous and lymphatic invasion. No neoplastic cells were present at the surgical margin. |
| **Case 5** | 20yo Azteca G** | Colic | Large, mixed echogenicity left paralumbar region mass. Heterogenous spleen. | None | **Euthanized**  Necropsy: large abdominal mass adhered to left kidney and great vessels. Metastasis to sumlumbar lymph nodes. |  |
| **Case 6** | 18yo QH  S | L testicle enlargement | Heterogenous and enlarged left testicle with discrete testicular mass | Unilateral L castration | **Discharged** 4 days after surgery (moderate scrotal hematoma)  **Euthanized** 3 years later for unrelated reasons | 10 mitoses observed in ten high powered 400x fields. Anisokaryosis and anisocytosis was marked. No neoplastic cells within surgical margin. |
| **Case 7** | 13yo TB  S | L testicle enlargement | Heterogenous and enlarged left testicle with 2 discrete hypoechoic masses. | Unilateral L castration | **Discharged** 5 days after surgery  **Euthanized** 1.5 years later due to acute weight loss, a caudal abdominal mass and bilateral pleural effusion  Necropsy: large abdominal mass adhered to vertebral column and left kidney. Metastasis to lymph nodes (sublumbar, mesenteric, sternal, hilar, tracheal and mediastinal), pulmonary pleura, pericardial sac and lungs. | 4 mitotic figures per 10 high power fields x 400. Vascular, lymphatic and tunic invasion. No neoplastic cells within the surgical margin, however there was a subjective increase in fibrous connective tissue. |
| **Case 8** | 12yo Fresian S | L testicle enlargement | Heterogenous and enlarged left testicle with loss normal architecture. Two discrete craniolateral masses. Chain of enlarged, heterogenous lymph nodes to the left of the terminal aorta, along the left iliac vasculature | Unilateral L castration | **Discharged** 4 days after surgery  **Euthanized** 9 months later due to acute weight loss and abdominal mass identified on ultrasound. | 26 mitoses observed in ten high powered 400x fields. Anisocytosis and aniskaryosis was moderate to marked. Vascular invasion. Neoplastic cells within the tunica albuginea. |
| **Case 9** | 20yo Lusitano S | R testicle enlargement | Heterogenous and enlarged left testicle with loss normal architecture. | Bilateral castration | **Discharged** 14 days after surgery (tuber coxae fracture and support limb laminitis)  **Euthanized** 3 years later due to acute weight loss, no further workup done | 11 mitoses observed in ten high powered 400x fields. Moderate anisocytosis and marked anisokaryosis. Vessel invasion. |

TNCC = total nucleated cell count. Yo = year old. G = gelding S = stallion. Crypt = abdominally retained testicle. QH = Quarter Horse. TB = Thoroughbred. R = right. L = left. LN = lymph node.

*= presumed gelding, unilateral crypt identified at necropsy

**=presumed gelding, bilateral crypt identified at necropsy
